# Early and sustained improvements of lung clearance index from two to sixteen weeks of elexacaftor/tezacaftor/ivacaftor therapy in patients with cystic fibrosis—a real world study

**DOI:** 10.3389/fphar.2023.1125853

**Published:** 2023-03-08

**Authors:** Dorothea Appelt, Gratiana Steinkamp, Sarah Sieber, Helmut Ellemunter

**Affiliations:** ^1^ Cystic Fibrosis Centre Innsbruck, Department of Paediatrics III, Medical University of Innsbruck, Innsbruck, Austria; ^2^ Clinical Research and Medical Scientific Writing, Schwerin, Germany; ^3^ STAT-UP Statistical Consulting & Data Science GmbH, Munich, Germany

**Keywords:** cystic fibrosis, modulator therapy, lung clearance index (LCI), ELX/TEZ/IVA, LUM/IVA, ventilation inhomogeneity, real-life, ppFEV1

## Abstract

Since the introduction of CFTR modulator therapies, longitudinal real-life data of lung clearance index (LCI) during treatment is scarce. In this single-centre, post-approval setting, we report data of 51 patients with different stages of lung disease, age 2–52 years with repeated measurements of forced expiratory volume as a percentage of the predicted value (ppFEV₁) and LCI after 2, 4, and 16 weeks of CFTR modulator treatment and at baseline. In 25 patients during elexacaftor/tezacaftor/ivacaftor (ELX/TEZ/IVA) treatment, significant improvements of LCI (median −1.4) and ppFEV₁ (median +8.3%) were observed after only 2 weeks, and were maintained after 4 and 16 weeks of treatment (LCI: -2.0, −2.2; ppFEV₁: +7.2%, +11.8%). We observed a significant correlation between LCI improvement at week 16 and lower baseline LCI. In 26 younger and healthier patients receiving lumacaftor/ivacaftor (LUM/IVA) treatment, no significant changes of LCI and ppFEV₁ occured. With ELX/TEZ/IVA, our data shows rapid, significant improvements of LCI and ppFEV₁ already after 2 weeks. Early LCI measurements can help to assess the patient’s response to this high-cost therapy.

## 1 Introduction

Cystic fibrosis transmembrane conductance regulator (CFTR) modulator combinations, such as lumacaftor/ivacaftor (LUM/IVA) and elexacaftor/tezacaftor/ivacaftor (ELX/TEZ/IVA), lead to a partial restoration of CFTR function, and clinical studies have shown improvements in several clinical outcomes (Heijerman et al., 2019; Middleton et al., 2019; Nichols et al., 2022). Real-life data regarding longitudinal improvements of ventilation inhomogeneity measured by lung clearance index (LCI) is rare, with one study in CF patients with severe lung disease <40% ppFEV₁ (per cent predicted forced expiratory volume in one second) with LCI measurements after 2 and 4 weeks and at baseline (Stylemans et al., 2022).

Graeber and colleagues recently published improvements of both ventilation inhomogeneity and structural lung damage measured after 8–16 weeks of ELX/TEX/IVA treatment (Graeber et al., 2022). That article prompted us to evaluate our own results of LCI measurements in patients treated with triple therapy. In contrast to other groups who reported single follow-up measurements or measurements only in children age 6–11 years (Zemanick et al., 2021; Graeber et al., 2022; Mall et al., 2022), we had assessed pediatric and adult patients longitudinally over 4 months, i.e. at baseline and after 2, 4, and 16 weeks of ELX/TEX/IVA treatment, as part of a routine monitoring programme of modulator efficacy. Our aim was to show the early changes in ventilation inhomogeneity in CF patients due to modulator treatment, measured by improvement of LCI. In addition, we analysed data from patients who had been treated with LUM/IVA before 2020. Here we report on repeated measurements of LCI and ppFEV₁ in patients of different age groups and at various stages of lung disease.

## 2 Materials and methods

In this single-centre real-world evaluation, we analysed data from all patients receiving ELX/TEZ/IVA (from 2020 to July 2022) or LUM/IVA (from 2013 to 2020) treatments with LCI measurements before and after beginning modulator treatment at the CF Centre Innsbruck. Patients were selected to start modulator therapy based on their genetics and deterioration over the last 3 years. We excluded patients receiving CFTR modulators in the context of clinical trials.

All measurements were part of a routine monitoring programme for newly prescribed CFTR modulator therapy using LCI, since 2006, as a surrogate marker for monitoring progression of CF lung disease (Ellemunter et al., 2010). At baseline and at weeks 2, 4, and 16 after commencing treatment, spirometry and multiple breath washout measurements were performed to determine the patient’s response to treatment. For the present evaluation, we collected data from our patient database which contains all results obtained during outpatient visits.

LCI was measured using multiple-breath washout (MBW), using nitrogen as the tracer gas. We used two different devices in our patient cohort: the EasyOne Pro® (NDD, Zurich, Switzerland) up to 2019, while the EXHALYZER D® device (Eco Medics, Duernten, Switzerland) has been used since 2017. Thus, all patients on ELX/TEZ/IVA and half of the patients from the LUM/IVA group were measured using the EXHALYZER D® device. All EXHALYZER D® measurements were reanalysed using the updated version of Spiroware® 3.3.1 (Eco Medics, Duernten, Switzerland) (Wyler et al., 2021). There is no validated correction function to address the signal correction error of the EasyOne Pro® device (Oestreich et al., 2022). The upper limit of normal for the two devices differ somewhat, with 7.0 for the EasyOne Pro® and 7.1 for the EXHALYZER D® (Fuchs et al., 2009; Wyler et al., 2021). Although two different MBW devices were used, all measurements throughout the 16 weeks of follow-up were performed with the same device in each patient.

Spirometry was measured in patients aged 6 years and older according to international standards (Quanjer et al., 2012). Since no extra multi-detector computed tomography (MDCT) scans of the chest were performed to assess modulator treatment response, the Bhalla scores (best possible score: 25) of the most recent examination before commencing therapy are displayed.

Ethics approval was obtained from the ethics committee at Medical University of Innsbruck (AN 2015-0227 353/2.5). Written informed consent was obtained from all patients and their legal representatives.

Results are expressed as medians and interquartile range. Absolute changes in ppFEV₁ and LCI from baseline were analysed using Repeated Measures ANOVA with *post hoc* tests (pairwise t-tests) (R Version 4.2.0, 2022). To evaluate the effect of severity of lung disease at baseline on subsequent treatment response, we divided each treatment cohort into two subgroups, with LCI and ppFEV₁ baseline values above or below the median, respectively. For a comparison of the two modulator groups with the whole patient cohort, the median ppFEV₁ was calculated from all patients aged 6 years and older (*n* = 153) treated at our centre in 2020.

## 3 Results

### 3.1 Patient characteristics

The patient characteristics of both treatment groups at baseline are summarised in Table 1. CFTR genetics differed between groups, since 13 of the 25 subjects on ELX/TEZ/IVA, but none on LUM/IVA, were heterozygous for the F508del CFTR mutation. Before receiving ELX/TEZ/IVA, six subjects had been treated with LUM/IVA and two subjects had been treated with tezacaftor/ivacaftor. Due to age restrictions in the licensing of ELX/TEZ/IVA, patients on triple therapy were older than LUM/IVA subjects and consequently had lower lung function, expressed as ppFEV₁ (median 53.8% vs. 76.6%) and higher LCI (13.1 vs. 9.6), respectively. Chest MDCT revealed more structural lung disease in the triple therapy group, i.e., median Bhalla scores of 12.5 vs. 18.0.

**TABLE 1:** Patient characteristics at baseline before CFTR modulator therapy with either Elexacaftor/Tezacaftor/Ivacaftor (ELX/TEZ/IVA) or Lumacaftor/Ivacaftor (LUM/IVA).

	ELX/TEZ/IVA			LUM/IVA		
	Median	Interquartile range	No. of patients	Median	Interquartile range	No. of patients
Patient characteristics						
Age [years]	24.2	17.9 to 32.7	25	19.0	9.3 to 28.5	26
Female: male [n]	19:6		25	17:9		26
CFTR delF508 mutation: homozygeous: heterozygeous [n]	12:13		25	25:0		26
Previous CFTR modulator therapy: LUM/IVA: Tezacaftor/Ivacaftor (TEZ/IVA)	6:2		25	0:0		26
MBW device for LCI measurements ExhalyzerD: EasyOne Pro [n]	25:0		25	12:14		26
Baseline Chest MDCT Score [Bhalla Score]	12.5	11 to 17	24	18.0	13 to 22	25
Lung clearance index (LCI) baseline	13.1	8.4 to 15.1	25	9.6	7.1 to 16.7	26
Percent predicted FEV₁ (ppFEV₁) baseline	53.8	44.5 to 85.7	25	76.6	51.8 to 91.1	20

All patients completed the 16 weeks of follow-up, except one patient, who died after week four of modulator therapy; the cause of death remained unknown despite an autopsy. Compared to the whole cohort at our centre (patients >6 years in 2020, without lung transplantation, median ppFEV₁: 84.3% *n* = 153), the two groups had more severe lung disease.

### 3.2 Response to ELX/TEZ/IVA treatment

**FIGURE 1:**
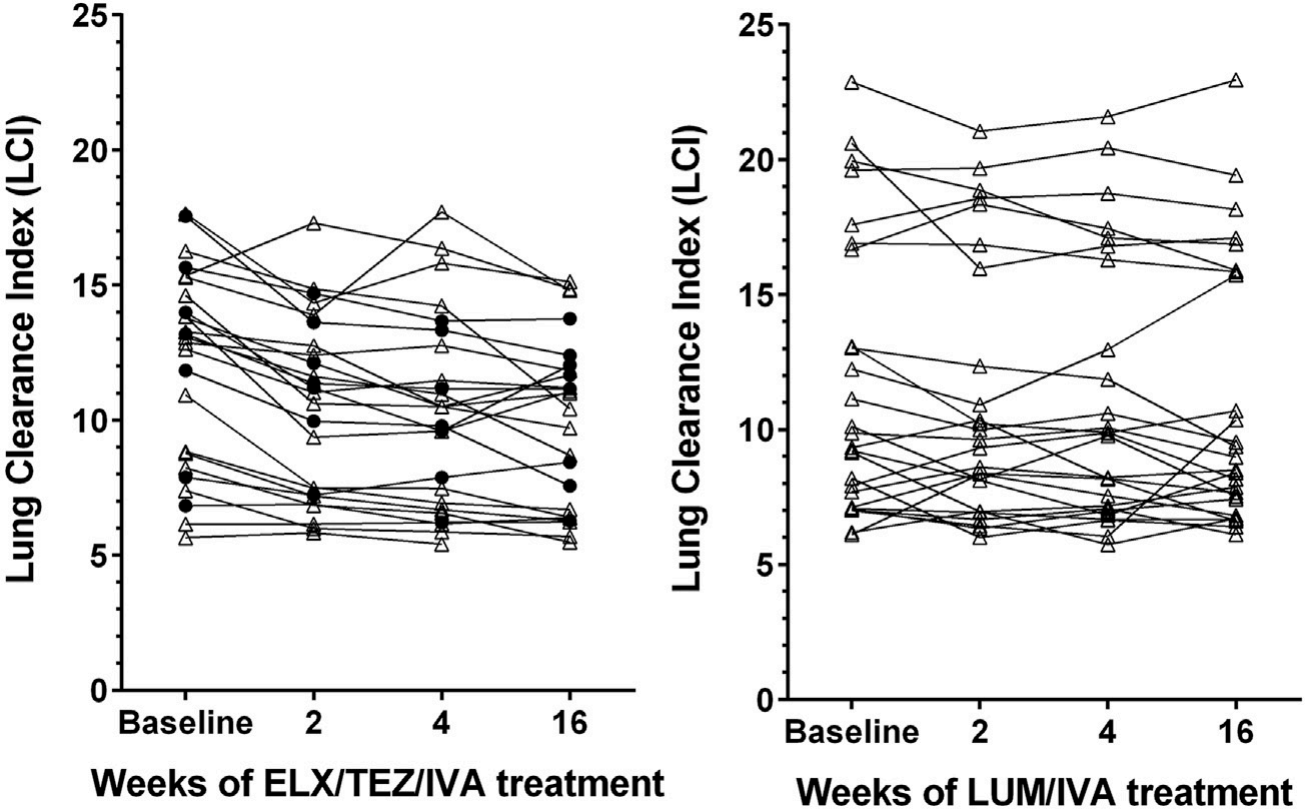
Line graphs depicting lung clearance index (LCI) in patients with cystic fibrosis after treatment with elexacaftor/tezacaftor/ivacaftor (left side, ELX/TEZ/IVA; *n* = 25) and lumacaftor/ivacaftor (right side, LUM/IVA; *n* = 26). Filled circles denote measurements in patients pretreated with another modulator, triangles denote measurements in patients who were modulator naïve before receiving ELX/TEZ/IVA.

Already after 2 weeks of ELX/TEZ/IVA treatment, significant (*p* < 0.0001) absolute improvements of both LCI (by −1.4) and ppFEV₁ (by +8.3%) were observed (Table 2). Median changes at weeks 4 and 16 showed consistent and clinically relevant improvements of both LCI and ppFEV₁. Thus, the benefits of ELX/TEZ/IVA treatment started early and were maintained up to the end of the observation period. Figure 1 depicts the LCI course of the individual subjects. Not only LCI of modulator naïve patients improved, but also those who had previously been treated with another CFTR drug, predominately LUM/IVA.

**TABLE 2:** Response to CFTR modulator therapy. ppFEV₁ and lung clearance index (LCI) at baseline, and absolute and relative changes at 2, 4, and 16 weeks after initiation of elexacaftor/tezacaftor/ivacaftor (ELX/TEZ/IVA) or lumacaftor/ivacaftor (LUM/IVA) in patients with cystic fibrosis. Six patients were not able to perform ppFEV₁ measurements due to age <6 years. Repeated measures ANOVA showed significant (*p* < 0.0001) benefits during ELX/TEZ/IVA treatment at each time point compared to baseline. Subgroup analyses revealed greater improvements in LCI and ppFEV₁ in patients with worse lung function, i.e. LCI above and ppFEV₁ below median, respectively.

	ELX/TEZ/IVA (2020 to 2022)			LUM/IVA (2013 to 2020)		
	Median	Interquartile range	No. of patients	Median	Interquartile range	No. of patients
Lung clearance index (LCI)						
Baseline	13.1	8.4 to 15.1	25	9.6	7.1 to 16.7	26
Absolute change from start of therapy to…						
week 2	−1.4	−2.3 to −0.6	25	−0.5	−1.5 to 0.9	26
week 4	−2.0	−3.0 to −0.5	25	−0.4	−1.4 to 0.7	26
week 16	−2.2	−3.9 to −1.1	24	−0.8	−1.7 to 0.5	26
week 16, baseline LCI below median	−1.7	−2.6 to −0.7	12	−0.4	−0.9 to 0.9	13
week 16, baseline LCI above median	−2.3	−4.4 to −2.0	12	−1.1	−3.1 to 0.2	13
Relative change (%) from start of therapy to …						
week 2	−12.7	−18.8 to −6.1	25	−5.0	−10.7 to 8.9	26
week 4	−15.4	−23.7 to −9.2	25	−4.2	−13.4 to 4.6	26
week 16	−16.2	−30.4 to −10.5	24	−6.7	−15.1 to 4.7	26
week 16, baseline LCI below median	−22.9	−30.9 to −7.2	12	−5.7	−12.8 to 10.5	13
week 16, baseline LCI above median	−15.4	−29.4 to −13.9	12	−9.0	−17.0 to −1.0	13
						
Percent predicted FEV_1_ (ppFEV_1_)						
Baseline	53.8	44.5 to 85.7	25	76.6	51.8 to 91.1	20
Absolute change from start of therapy to…						
week 2	8.3	4.0 to 16.1	25	0.9	−4.4 to 5.6	20
week 4	7.2	5.2 to 15.8	25	−0.3	−3.9 to 9.5	20
week 16	11.8	6.6 to 15.3	24	2.8	−2.9 to 7.2	20
week 16, baseline ppFEV₁ below median	12.2	9.0 to 14.29	12	5.4	1.8 to 8.2	10
week 16, baseline ppFEV₁ above median	10.1	5.8 to 16.3	12	0.1	−6.3 to 3.9	10
Relative change (%) from start of therapy to …						
week 2	15.0	8.1 to 28.6	25	1.5	−5.2 to 8.3	20
week 4	15.7	6.7 to 28.7	25	−0.3	−4.8 to 11.9	20
week 16	19.9	10.0 to 28.3	24	4.2	−2.2 to 13.7	20
week 16, baseline ppFEV₁ below median	28.6	18.9 to 41.0	12	11.4	4.0 to 15.4	13
week 16, baseline ppFEV₁ above median	12.1	6.6 to 21.5	12	−0.1	−6.7 to 4.4	13

**FIGURE 2:**
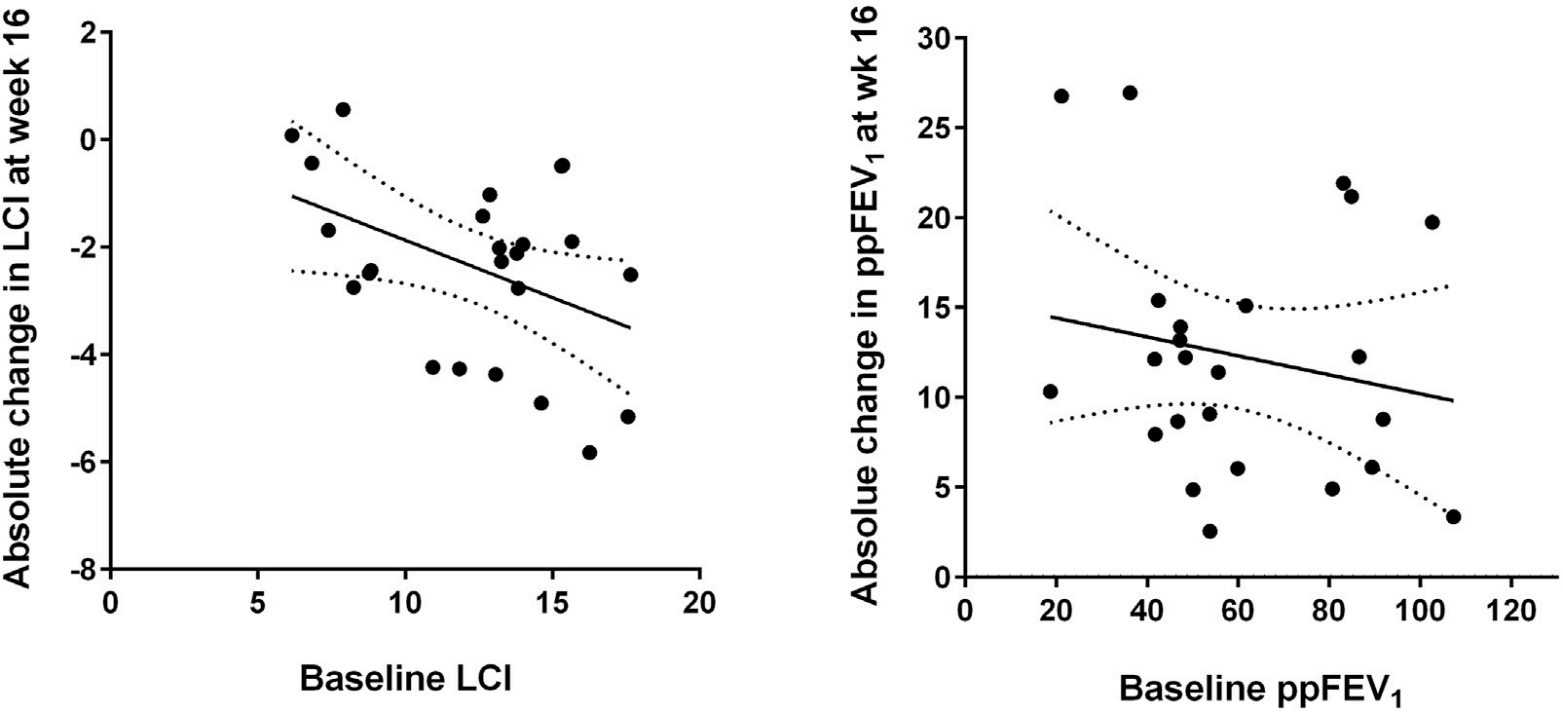
Absolute changes in lung clearance index (LCI, left) and per cent predicted forced expiratory volume in one second (ppFEV₁, right) after 16 weeks of elexacaftor/tezacaftor/ivacaftor (ELX/TEZ/IVA) treatment compared to the respective baseline values. The best-fit linear regression lines and their 95% confidence bands are displayed (see text for correlation coefficients).

Analysis of subgroups with more or less severe lung function impairment revealed that disease severity was associated with treatment response, since patients with worse baseline LCI (above the median of 13.1) showed a larger median improvement in LCI after 16 weeks than subjects with better baseline LCI values (Table 2). Figure 2 shows that the improvement in LCI after 16 weeks of treatment was significantly correlated with baseline LCI values (r = −0.431, *p* = 0.037), while there was no association of change in ppFEV₁ at week 16 with baseline ppFEV₁ (r = −0.188, *p* = 0.380).

At week 16, the changes of the two parameters LCI and ppFEV₁ compared to baseline showed a trend to correlate with each other, but without statistical significance (r = −0.375, *p* = 0.071). Change in LCI was neither associated with baseline ppFEV₁ (r = 0.088, *p* = 0.683) nor with baseline MDCT Bhalla score (r = 0.119, *p* = 0.587).

### 3.3 Response to LUM/IVA treatment

The 26 younger and healthier patients who received LUM/IVA between 2013 and 2020 experienced no significant changes in LCI (Figure 1), although the median LCI declined by −0.8 at week 16 compared to baseline (Table 2). Absolute change of ppFEV₁ also showed no significant benefit from LUM/IVA treatment at weeks 2–16. The only subgroup which experienced a detectable response to LUM/IVA were patients with lower initial ppFEV₁ (below 76.6%), with a median ppFEV₁ increase of 5.4% at week 16.

## 4 Discussion

Our data from a real world, post-approval setting showed clinically relevant and statistically significant improvements of ppFEV₁ and LCI already after 2 weeks of ELX/TEZ/IVA treatment, with benefits maintained 4 and 16 weeks after the first dose. There was a correlation between baseline LCI and the improvement of LCI after 16 weeks of therapy. We evaluated patients between 8 and 52 years of age with predominantly moderate lung disease, as reflected in a median ppFEV₁ of 53.8%. Complementing other studies, our patients had three control visits within the 4 months after initiating CFTR modulator therapy to get a clearer picture on the response to treatment.

The recent article by Graeber et al. reported LCI improvements in 45 patients heterozygous for F508del (by −2.4) and 46 homozygous for F508del (by −1.4) aged 12 years and older with ELX/TEZ/IVA treatment (Graeber et al., 2022). Only one measurement was performed 8–16 weeks after commencement of treatment, so the situation within the first weeks of treatment remains unknown. Two other studies in children 6 through 11 years described longitudinal data for LCI before and during treatment with ELX/TEZ/IVA (Zemanick et al., 2021; Mall et al., 2022). The authors observed significant improvements in LCI of −2.3 and −1.7, respectively, and ppFEV₁ (+11.0 and +10.2%) after 24 weeks of treatment compared to baseline. Depicting the longitudinal course of LCI during ELX/TEZ/IVA treatment in adults, only one paper describes measurements after 2 and 4 weeks with improvements of both LCI and ppFEV₁ by median LCI -0.6 and −1.4, and median FEV₁ +6% and +3% in older patients with severe lung disease, i.e. an ppFEV₁ below 40% predicted (Stylemans et al., 2022).

Regarding LUM/IVA treatment and LCI, four clinical trials showed significant LCI changes compared to baseline, with two trials focusing children from 6 to 11 years and two trials analysing patients >12 years (Milla et al., 2017; Ratjen et al., 2017; Shaw et al., 2020; Graeber et al., 2021). In single measurements obtained at 24 or 52 weeks after start of LUM/IVA, improvements of LCI between −0.8 and −1.1 were observed, whereas no change in ppFEV₁ compared to baseline was detected (Milla et al., 2017; Ratjen et al., 2017; Shaw et al., 2020; Graeber et al., 2021). Our data also shows small improvements in median LCI during LUM/IVA treatment mainly in patients with a higher LCI at baseline, but to a much lesser degree than during triple therapy. 2 weeks after beginning LUM/IVA treatment 16 of 26 patients had an improved LCI, compared to 21 of 25 patients with ELX/TEZ/IVA treatment. The cohort treated with LUM/IVA showed a younger age with preserved lung function, which could explain the smaller but still discernible effect on LCI.

Our work does have several limitations. First, it is a single-centre, not controlled or blinded study. Second, we used two different MBW devices with non-interchangeable results. However, since each patient used the same measurement device throughout the study period, we regard the intrapatient differences as suitable to represent treatment effects. Third, Exhalyzer D software update was implemented in September 2019 (Spiroware® 3.3.1) as a reaction to technical progress. To avoid the bias of overestimating LCI improvement, we used an updated software version to reanalyse all measurements of Exhalyzer D before September 2019. Fourth, the treatment groups receiving ETI or LI therapy were not comparable, since the latter were healthier and might have less room for improvement from CFTR modulator therapy.

A strength of this work is that as no limitation concerning severity of lung disease was defined as exclusion criteria, we can show data from a diverse CF collective, and we also included patients with normal lung function and with baseline LCI below 7. Therefore, we could analyse the treatment response in patients with less and more severe lung disease. We found that subjects with worse baseline LCI experienced a greater benefit from ELX/TEZ/IVA at week 16 than those with less ventilation inhomogeneity. Moreover, this is the second published work after Stylemans et al. with longitudinal real-world LCI data in adults with measurements already after 2 weeks of ELX/TEZ/IVA treatment (Stylemans et al., 2022).

In conclusion, the present results from our routine monitoring programme provide further detail on the rapid treatment response during CFTR modulator therapy. If in doubt whether a patient responds to ELX/TEZ/IVA therapy, measuring LCI and ppFEV₁ within a few weeks after commencing modulator therapy can provide important information on the efficacy of this high-cost therapy. Our data suggest that measuring baseline LCI could help in estimating which patients might benefit the most from CFTR modulator therapy. Compared to the older drug LUM/IVA, the improvements induced by ELX/TEZ/IVA were substantial and clinically relevant. Further studies should evaluate the long-term effects of modulator therapies on ventilation inhomogeneity, including the effectiveness after reduction of routine symptomatic CF treatments.

## Data Availability

The raw data supporting the conclusions of this article will be made available by the authors, without undue reservation.

## Abbreviations

CF, cystic fibrosis; CFTR, Cystic fibrosis transmembrane conductance regulator; LCI, lung clearance index; MBW, multiple breath washout; MDCT, multi-detector computed tomography; ELX/TEZ/IVA, Elexacaftor/Tezacaftor/Ivacaftor; LUM/IVA, Lumacaftor/Ivacaftor; ppFEV:, per cent predicted forced expiratory volume in one second.
